# Interventions to enhance newborn care in north-West Ethiopia: the experiences of health care professionals

**DOI:** 10.1186/s12884-022-04669-0

**Published:** 2022-04-15

**Authors:** Bizuhan Gelaw Birhanu, Johanna Mmabojalwa Mathibe-Neke

**Affiliations:** 1 UNICEF Ethiopia Country Office, Community Newborn and Child Health Specialist, Addis Ababa, Ethiopia; 2 Department of Health Studies, College of Human Sciences, University of South Africa (UNISA), Pretoria, South Africa

**Keywords:** Ethiopia, Factors, Newborn, Primary healthcare

## Abstract

**Background:**

The provision of optimal and quality services during labour, delivery and in the early neonatal period is highly required to accelerate the reduction of neonatal deaths and improve the quality of life of newborns. The availability of competent health professionals and the essential medicines and supplies in the health facilities are compulsory. Cost-effective interventions exist to prevent more than 80% of all newborn deaths. However, an unacceptably high number of newborns are dying in the study area, and much is not known about the main contributing factors in primary healthcare settings. This study aimed to explore and describe the quality of care provided to newborns in the primary healthcare units.

**Methods:**

Qualitative exploratory and descriptive design was employed. Focus group discussions were held with 26 participants (11 health workers and 15 health extension workers) in three *woredas* in the West Gojjam zone, Ethiopia. Health workers and health extension workers were purposely selected. Thematic analysis was undertaken.

**Results:**

The primary healthcare facilities play a major role in the provision of essential services for newborns in the critical periods, including during labor and birth, immediately after birth and in the early postnatal care period. Resuscitation of birth asphyxia, prevention of hypothermia, initiation of breastfeeding, application of tetracycline, vitamin k injection, weighing babies and chlorhexidine application were identified as immediate essential intervention for the newborns. However, these interventions are hampered by factors such as lack of adequately trained staff & hands-on skills; weak referral linkage; stock-out of essential medicines and supplies and poor quality for early postnatal care home visits.

**Conclusions:**

In order to enhance the quality of newborns healthcare provision, the health-systems constraints at health centres and heath posts level should be fixed to provide the required services for newborns. This requires allocation of adequate resources to tackle health facilities readiness related bottlenecks, such as the frequent stock out or lack of essential supplies, equipment, and medicines, lack of proper space for the service provision, not systematic replenishing of the revised job-aids and maintenance of medical equipment, poor infection prevention including water and sanitation in the maternity wards and newborn corners.

## Introduction

Ethiopia has a population of about 103 million in 2021. It is the second most populous country in Africa. The Amhara region is one of 11 administrative regions with a population of 22,535,997. In the Amhara regional state of Ethiopia there are eleven zones, and West Gojjam (Mirab Gojjam) is one of the zones that belongs to this region. It has a total population of 2,686,968 as per the 2007 census projection conducted by the Central Statistical Agency of Ethiopia [1].

Neonatal conditions in Ethiopia account for 55% of under-5 deaths, with 33 neonatal deaths occurring for every 1000 live births (LBs). About 108,766 newborns die every year in Ethiopia. In the Amhara region, the burden of neonatal mortality is much higher than the national average; 71.9% of under-5 deaths are contributed by neonatal conditions in the first 28 days, with 46 newborns dying from every 1000 live births or about 32,136 newborns dying every year. The pattern of neonatal mortality is similar in all zones of the Amhara region, including the West Gojjam Zone. That is why the researchers selected the West Gojjam zone in the Amhara region as the study area for this research [2].

During delivery, as such, health professionals should be responsive to safe newborn lives, with the aim of preventing morbidity and mortality [3]. As a result, if the essential quality of services is provided for the mothers and their babies during labour and delivery and in the early postnatal period particularly within the 24 h, it is expected to contribute to a significant reduction of newborn deaths [4]. Wardlaw et al. reported a huge variation on the utilization of services by pregnant women and newborns and quality of services provision by health system to pregnant women and newborns, as per the summary of United Nations Children’s Fund (UNICEF) report [5]. Furthermore, UNICEF 2014 highlighted that, quality of care is not offered for the newborns and mothers who are already visiting the health system. And that many mothers and newborns miss out key interventions that can save their lives [4]. The assessment done on newborn care in Indonesia, Lao People’s Democratic Republic and the Philippines in 2014 reported that within primary healthcare and referral level health facilities, newborn care services provision is found to be poor in quality. It is mainly related to the incompetency of health professionals [6].

As stated by Kruk, et al. 2018, and Lawn et al. 2014, most newborns lives can be saved if the health facilities provides high-quality care by ensuring the availability of essential medicines and commodities, compliance with evidence-based clinical interventions and practices, adequate infrastructure and supplies to ensure infection prevention and control, competent, motivated staff and solid documentation and use of information [3, 7]. According to Knippenberg, et al., essential interventions contributing for the reduction of neonatal morality are not dependant on technologies or supplies; rather on the adequate skills that the health care providers should be competent on at all levels [8]. According to every newborn action plan [9] each country is expected to reduce their respective neonatal deaths up to the level of ten or less per 1000 live births by 2030.

According to the 2021 Amhara region health bureau annual report, 65% of deliveries were attended by skilled birth attendants in 874 health centres, whereas the remaining 35% of deliveries were attended by 98 hospitals. In the same pattern, 70 and 30% of births are attended by 108 health centers and 8 hospitals respectively in the West Gojjam zone [10, 11]. It shows primary healthcare shares a huge burden of care during birth and in the early postnatal period. Even though the health extension workers are providing antenatal care, facilitating referral linkage for delivery, early postnatal home visit, and treating sick newborns and young infants at the health post level, currently, they are not supposed to attend delivery.

As stated in the Health Sector transformation Plan (HSTP II), reducing newborn mortality is a top priority of the Ethiopian Government and it is aiming to reduce neonatal mortality from 33 per 1000 live births in 2019 to 21 in 2024/2025 [12]. Consequently, priority initiatives are identified and clearly stipulated in this overarching document. The accessibility of services to the community by expansion of primary healthcare services has been increased, and efforts have been made to improve the quality of care in the health system. However, it has been impacted by uneven distribution of health resources, suboptimal quality of care, low care seeking behavior of communities, and shortage of essential health commodities and equipment at service delivery points [12, 13]. These were identified as the remaining key challenges contributing to high rates of neonatal mortality in Ethiopia which requires a consolidated effort.

Cost-effective interventions exist to prevent more than 80% of all newborn deaths [14]. However, an unacceptably high number of newborns are dying in the study area, and much is not known about the main contributing factors in primary healthcare level.

The key findings of this study are expected to improve the body of knowledge on the provision of high-impact interventions and the quality of care for newborns in the primary healthcare setups.

### Objective

This study aimed to explore and describe the quality of care offered to newborns by frontline healthcare providers in primary healthcare units in the North-West of Ethiopia.

## Methods

### The research approach and design

Qualitative exploratory and descriptive design was employed [15, 16]. Focus group discussion (FGD) was selected as one of the techniques that produce data in collective space in such kinds of expert groups [17, 18].

### Population of the study

Three *woredas (*districts*)* namely Bahir Dar Zuria, Mecha and South Achefer in the West Gojjam zone, North-West of Ethiopia were selected purposely for the study. West Gojjam is one of the 11 administration zones in the Amhara regional state and consisting of fifteen *woredas*. In these selected *woredas*, three health centres (HCs) and four health posts (HPs) were selected purposely. The targeted population was health workers (HWs) in HCs who were working in maternity ward (delivery and early post-natal) and under-five clinic and all health extension workers (HEWs) who were working in the HPs in selected heath facilities.

### Data collection

Written consent was obtained from each participant including audio recording of their responses. Participation was voluntary. The focus group discussions (FGDs) were held separately for the HCs and HPs staff. Twenty-six participants were interviewed through FGD in seven rounds of discussions. Health workers and HEWs who were working in the maternity wards and under-five clinics participated in the FGD’s. The principal investigator specified the objectives and information needs of the FGD and took the responsibility of moderation to facilitate the discussion, probing, and encouraging all the members to participate. The assistant moderator was taking notes and the discussions were held at their respective private room in the health facilities. In addition to note taking, the discussions were recorded with audio-tape. After each session of FGDs, the audio-recorded information and note recorded were checked for their consistency.

### Data analysis

Data analysis was initiated within the data collection process. All audiotape records were transcribed into *Amharic* and the 44 pages’ *Amharic* transcription was directly translated into English by linguistic expert. Thematic analysis was employed with different stages, familiarizing the data, generating initial codes, searching for themes, reviewing the themes, defining and naming the themes and producing the report [19, 20]. Overall, the qualitative analysis was done manually.

### Ethics approval and consent to participate

This study was conducted under the ethical principles of the Declaration of Helsinki. Ethical clearance was obtained from University of South Africa (UNISA) Research and Ethics Committee (Ref no: HSHDC/489/2015). The Amhara regional health bureau and West Gojjam Zone provided permission to conduct the study. Participants privacy and the confidentiality of information given were maintained throughout the study. Information on the study’s purpose, procedures, risks, burdens, and benefits as well as confidentiality was provided. Written informed consent was obtained from each study participants before the discussion and confidentiality of information was maintained throughout the study. Field notes and tape-recorded voices were kept properly and the health facilities and health professionals’ unique personal identification was anonymized in the report or shared [21].

### Trustworthiness

The FGDs interview guide was pre-tested to ascertain the credibility. Likewise, to ensure dependability, the audio data and transcribed data both in English and *Amharic* kept for further verification. The data collection process in the field and data analysis procedure was documented. In addition, FGDs participants’ voice and speech were quoted and included as part of the report as a strategy of ensuring conformability. Transferability was confirmed by providing a thorough explanation of the methods of the data collection and its tool, the types of healthcare providers, the steps and the procedures applied for the analysis and presentation to support the major quantitative findings [22, 23].

## Results

Amongst the four FGDs, three of them were held with health providers who were working in the respective HCs; and the rest of the four discussions were conducted with HEWs at HPs level. Participants in this FGDs were identified and reported as per their group and participant number given during each FGDs (Tables 1 and 2). The FGDs key finding is presented and discussed based on the identified major themes and sub-themes Due to the level of care difference between the HCs and HPs, the emerged themes are reported discretely.

**Table 1:** FGD participants among HWs in HCs in West Gojjam Zone, Ethiopia

Focus group	Number of participants	Identification of participants in their group	Unique identification number for each participant
Group 1	4	Group1- Participant 1–4	G1/P1–4
Group 2	3	Group2- Participant 1–3	G2/P1–3
Group 4	4	Group 4- Participant 1–4	G4/P1–4
Total	11

**Table 2:** FGD participants among HEWs in HPs in West Gojjam Zone, Ethiopia

Focus group	Number of participants	Identification of participants in their group	Unique identification number for each participant
Group 3	6	Group 3- Participant 1–6	G3/P1–6
Group 5	3	Group 5- Participant 1–3	G5/P1–3
Group 6	3	Group 6- Participant 1–3	G6/1–3
Group 7	3	Group 7- Participant 1–3	G7/P1–3
Total	15		

### Interventions and platforms for newborn care services

#### Experiences of the HCs on neonatal health care services

The immediate and essential newborn care services are provided in HCs for the newborn such as resuscitation if she/he develops birth asphyxia, initiated breastfeeding, application of tetracycline in the eyes, vitamin k injection, weighing and chlorhexidine application in the cord (Table 3).

**Table 3:** Major themes and sub-themes of HC staff participants’ responses in West Gojjam Zone, Ethiopia

Major themes	Sub-themes
Experiences of the HCs on neonatal health care services	Chlorhexidine jel (4%) application for cord care
	Management of preterm labour
	Management of birth asphyxia
	Kangaroo mother care
	Early PNC for the mothers and newborns
Quality of neonatal healthcare services	Availability of material resources
	Competency of HCs personnel
Referral linkage	Linkage for early PNC home visit

#### Chlorhexidine (4%) application for cord care

In line with the experience in the use of chlorhexidine (4%), “Yimserach jel” the local brand name, was discussed with HWs. Thus, “Yimserach jel” was available in the HCs. As per the ministry of health recommendation, the application has started in HC after delivery and the mother take away the remaining to apply at home.

G4/P1: *“It is available. We [HC staffs] apply once and demonstrate to them then they apply the remaining 6 days.”*

#### Management of preterm labour

All the HCs discussants agreed that, they are not equipped to provide intramuscular dexamethasone or other corticosteroids for pregnant woman at risk of preterm birth; and guideline is not available in their respective health facilities. Consequently, if the HCs are confronted with preterm labour, referring the pregnant woman to the higher-level facility is the usual practice.

G2/P3: *“No service [for preterm labour]. There is some concept during in some training, but not practically available. And dexamethasone service is not available. We refer to higher facility if preterm labour occurred.”*

G4/P1: *“Management of preterm labour guideline is not available, we [HC staff] haven’t trained yet.”*

#### Management of birth asphyxia

The HCs group discussion participants revealed that they have relatively adequate knowledge and received training on the management of birth asphyxia to save the lives of newborns immediately after birth. However, most of the HCs staff complained that the narrow space in the delivery room; shortage of supplies for resuscitation; misappropriate use of supplies; and limited competency since the HCs staffs are not often practicing the skills mainly due to the limited number of case were some of the factors mentioned by the HCs staff which was affecting the resuscitation process.

G2/P3: *“ … shortage of material supply, misappropriate use of some plastic materials, technical problems … poor skills of the HWs, practical training should be mandatory.”*

G4/P1: *“The class is very small to resuscitate the baby and no table for resuscitation … there is no resuscitation section/room … the case [newborn with birth asphyxia] is not present in the previous 6 months.”*

#### Kangaroo mother care (KMC)

In the discussion with the HCs staff, often KMC was only initiated in cases of referral of very /low preterm or low birth weight newborns to hospitals. Otherwise, cases were not admitted at HCs for KMC services. In addition, initiating KMC at HC and linking to the HP was not a common practice as well. Most of the HC staffs confirmed that, the HCs are not ready to provide the required KMC services since the existing rooms are already overstretched, or lack of room and poorly equipped with beds and the required supplies.

G4/P1: *“We [HC staff] initiate KMC and then refer them to the higher facility [hospitals]. It is advantageous if KMC section has one room independently … there is no defined room for KMC service. There is no on-job training and no referee guidelines to practice KMC.”*

#### Early PNC for the mothers and newborns

All the HC discussion participants agreed that, they were experiencing early discharge than the recommendations to stay the mother and baby for the 24 h after delivery in their respective health facilities. Among the frequently mentioned reasons for early discharge were lack of dedicated space and beds for PNC in their respective HCs. In addition, once the mother gave birth the family members and the accompanies consider that there is no problem after birth and they want to go and practice some traditional celebration at home with their families and neighbours.

G2/P3: *“Early discharge takes place because of absence of enough space or room … we [HC staffs] discharge them within six hours after birth. There is also understanding problem among the community, they think that as if there is no problem after mothers give birth, and they ask immediately for discharge after birth”.*

### Quality of neonatal healthcare service provision

Regarding the quality of the neonatal healthcare provision at HC level, due to suboptimal availability of trained human resources, material resources and essential medicines and supplies, most of the HC participants agreed that the service provided for the newborns is not as high as expected quality standards. Nevertheless, some HC participants argue that the HCs are trying their best to provide the quality of health care services for the newborns.

#### Availability of material resources

The participants agreed that, even though there was a positive trend, sometimes, health facilities were still experiencing stock-out of essential supplies, medicine and job-aids. Adequate or dedicated space for KMC and early PNC was not available in the HCs.

G2/P2: *“Yes, adequate medicine and job-aids are present, only last time there was shortage. Within 2 years of my experience in this HC there was shortage of Ampicillin for some three months.”*

#### Competency of HCs personnel

HCs staffs claimed that the quality of neonatal health care services at HC level was sub-optimal and the consistency of the services was not always maintained at all the times. Lack of trained human resources, newborn health reference guidelines to up-to-date the knowledge and practice, and motivation were some key factors affecting the quality of service provision.

G1/P2: *“There is shortage of trained staff, we can’t conclude there is quality service delivery.”*

### Referral linkage

Though the referral link not strong enough, each level of care across the primary healthcare system is also connected to referral linkage.

### Linkage for early PNC home visit

There was no a strong mechanism established at HCs level to inform the HEWs at HPs level about the birth occurring at HCs level for their early home visits for PNC. In fact, some of the HCs staffs were sending a green colour notification card to the HEWs at HPs to continue the PNC and other essential services for the newborn and the mother.

G2/P3: *“There is a problem of reaching to HEWs … some husbands take the green notification card [from the HC during discharge] and ignore to give to HEWs. The card contains time of birth, infants’ weight. HEWs can’t get this information if the card did not reach them. When we ask HEWs to check whether they got those cards they replied that as they do not have any information, and we give them information again for the 2nd time.”*

### Experiences of the HPs on neonatal health care services

HEWs’ who are working in the HPs, the first level of care in the Ethiopian health system. HEWs participants revealed that, most of the community based newborn care services initiated at HP such ANC, facilitating health facility delivery and early PNC for essential newborn care (Table 4).

**Table 4:** Major themes and sub-themes of HEWs participants’ responses in West Gojjam Zone, Ethiopia

Major themes	Sub-themes
Experiences of the HPs on neonatal health care services	Neonatal intervention across the continuum of care
	Facilitating transportation services for pregnant women
	Early PNC home visits
	Counseling on thermal care and breastfeeding
	Birth notification
	Chlorhexidine jel (4%) application
	Detection of preterm or low birth weight babies

#### Antenatal care screening

HEWs confirmed that they were mainly engaged in the identification of pregnant women in their respective community, followed by provision of the antenatal care (ANC) services at HP level. They are also referring the pregnant women to the nearby HCs for the additional ANC services.

G7/P2: *“We [HEWs] tell her danger signs may occur during pregnancy such as: things related to anaemia and blood pressure, we counsel them to get follow-up service and refer them if the problem is somewhat complex … , because there is a pregnant woman waiting home in the HC, we also advise her to stay there when the expectant mother enter her 9th month”.*

#### Facilitating transportation services for pregnant women

Most of the HEWs agreed that they were trying their best in connecting the pregnant women as soon as labour is initiated or anticipated to get the ambulance transportation services from their home to HC or hospital to facilitate professional assisted delivery. Because of this facilitation, HEWs believe that the coverage of delivery in the HCs or hospitals has significantly increased when it compared with the previous years.

G3/P1: *“During the pregnant women conference we give them [pregnant women] the ambulances phone address to call when labour occurred.”*

#### Early PNC home visits

The experience on provision of early PNC was varied among the HEWs participants. Some of the homes either did not receive or received a delayed PNC visit by HEWs; even some of the women didn’t get the PNC visit at all. Majority of newborns will not get the key lifesaving interventions and essential newborns care in the recommended period by HEWs, particularly for those mothers who gave birth at home and early discharged from the health facilities.

G7/P3: *“There were 109 births and we made home visit for PNC between 3-7 days, there were mothers who didn’t get the home visit for PNC.”*

G6/P2: *“When we are going for other duties [in the village] we don’t carry nothing because of our attention is on the other job.”*

The study also explored the existing birth notification or communication system in placed to carry-out the home visits for PNC by HEWs. However, some common mechanisms exist to notify births, but there are no a clear and standard pathway for timely notification of births. Occasionally, HEWs were getting report or information from the community health works (Women Development Armies), and rarely they were receiving a message from the delivered mother by themselves like ‘come and see me’. Overall, the timeline range receiving notification by HEWs is about 2–7 days from the onset of delivery.

G7/P2: *“We know most of pregnant women are giving birth at health centre; the midwife writes a letter for us. There is a paper the mother supposed to gives to us. … but sometimes we get the notification letter at 45 days when they come for vaccination.”*

#### Health information on thermal care and breastfeeding

During home visits, HEWs agreed that they are providing counselling services to delay bathing of the neonate, assessing the feeding condition of the neonate following with counselling of the mother especially for exclusive breastfeeding up to 6 months; and reminding the schedule of the immunization at 45 days of birth.

G7/P3: *“We [HEWs] check also her [mother] feeding situation, breastfeeding status of the neonate.”*

#### Chlorhexidine (4%) application

In the discussion with the HEWs participants’ chlorhexidine (4%), “Yimserach jel”, was lacking in HPs despite the current policy does not allow HEWs to attend delivery at HP level. However, still a significant number of mothers gave birth at home where infection prevention is a concern. Thus, the role of HEWs are limited to checking the application of “Yimserach jel” during home visits for those mothers who gave birth at HC or hospital and if they received the jel.

G3/P1: *“They gave us [HEWs] sample but not available now. It is available at HC. Many mothers told me that they took it from HC and applied to their newborn’ umbilicus.”*

#### Detection of preterm or low birth weight babies

In addition, most of the time HEWs were not carrying the required tools for PNC home visits such as weight measurement scale, thermometer and timer. As a result, the weight of the newborns is not taken and assessed for birth weight especially for newborns at home delivery.

G3/P4: *“All of us are not practicing checking their weight and count breathing after we make follow-up of infants, but only registering them … .”*

## Discussion

Immediate drying and additional stimulation or resuscitation for birth asphyxia, prevention of hypothermia including skin-to-skin contact in the first hour of life, late cord clamping after 1–3 min, initiation of breastfeeding, application of tetracycline, vitamin k injection, weighing the babies, and chlorhexidine application are the immediate essential newborn intervention which are provided at the health centres. In addition, during early postnatal home visits, prevention of hypothermia including delay bathing of the newborn, optimal breastfeeding, weighing the babies and chlorhexidine application were often recommended to be practiced by HEWs. This study found that, as per the perception of the frontline healthcare providers, the quality of care provided to newborns in the primary healthcare units was affected by the non-availability of material resources and limited skill set of the HCs staff. The findings of this study is supported by Islam et al. 2015 [24] on the study conducted to understand the views of healthcare professionals and their patients on the quality of care provided for newborns and their mothers in healthcare facilities in Bangladesh. It highlighted that inadequate healthcare providers and supplies; non-adherence to job-aids or service delivery standards; staff not adequately trained; and inadequate mentorship or supportive supervision from hospital clinicians were some of the bottlenecks that were affecting the quality of healthcare provision for newborns and their mothers.

Regardless of the World Health Organization (WHO) and national recommendations [25–27] on the timing of discharge, at least 24 h after birth from the health facility for an uncomplicated vaginal birth; this study found that, HCs were not retaining mothers and newborns for PNC at least 24 h after delivery. This is due to the limited space in the health centre, and the preference of women’s families to go home immediately after delivery. This was a great missed opportunity for the mother and the newborn to receive the early PNC in the critical periods, since majority of neonatal mortality occurs within the first 48 h after birth. Similarly, as stated by Tiruneh, et al. 2020, on the determinants of postnatal care utilization in Ethiopia, health service-related factors were reported as a significant determinants for use of postnatal care [28]. As continuum of care, the Ministry of Health Ethiopia guided the HEWs should carry-out early postnatal home visits [29]; however, the coverage of early PNC home visits is very low and the quality of visits was poor or suboptimal. This is in line with the study conducted regarding postnatal home visitation by McPherson and Hodgins 2018 in the 12 countries, the provision of postnatal care within 48 h of after birth following home birth is below 10% in most of the countries [30]. Another qualitative study done on the early postnatal home visits in two regions of Ethiopia, reported that, some inaccessible areas did not receive visits [31].

As stated by American Academy of Pediatrics 2011, a newborn baby who fails to breath at birth, shall be quickly dried, stimulated and resuscitated with bag and mask, to help the baby to breathe in 1 min, “golden minute”. Furthermore, ventilation with bag and mask is the most important and effective way to open the lungs of the baby with air. Delay in ventilation may result in preventable death or brain damage [32]. In this study the HWs believed that they have basic knowledge and skills to manage birth asphyxia, but they considered that the resuscitation process is impacted by lack of adequate space, shortage and misappropriate use of supplies, and they were not confident enough on their skill set due to the limited exposure to the cases of birth asphyxia. In line with this, the study done in Tanzania by Haug et al. 2020 on the video analysis of newborn resuscitations after simulation-based helping babies breathe training [33] noted that the actual newborn resuscitation practice deviated from the recommendation such as ventilation was delayed and frequently interrupted. In another qualitative study in Tanzania [34] on factors affecting effective ventilation during newborn resuscitation, the readiness’s of the ventilation supplies in delivery room, working as a team and repeated training to improve the competency skills found to be the trigger factors for the health care providers to provide and practice of the immediate newborn care practice.

It is an important intervention that is aiming to ensure the thermal care for both preterm and low birthweight newborns. KMC should be initiated in the health facilities and the newborn should stay in the KMC for 24 h [27, 35]. However, in this study, the HCs were mainly exercising KMC services as prereferral intervention to hospital or due to the poor quality and delayed PNC home visits, HPs staff were not detecting the preterm or low birth weight babies at community level. Similarly, as per a systematic review to assess the bottlenecks and facilitators on the implementation of KMC services across in the health system; both healthcare workers and their health facilities face unique barriers to implementing KMC [36].

In Ethiopian context, regardless of the place of delivery, a-daily chlorhexidine (4%) application for 1 week is recommended [29]. The application of chlorhexidine (4%), “Yimserach jel” on the umbilical cord stump of the newborn initiated immediately after birth and daily application for the following 6 days at home. In this study, the response of the HCs staff was consistent with the national recommendation. Chlorhexidine was included as one of the components in the community based newborn care implemented by HEWs [37], however, this study found that, in the early phases of “Yimserach jel” introduction, it was available at HP level, later, often “Yimserach jel” was not refilled and available at HPs level. Consequently, mothers who gave birth at home their newborns won’t get Yimserach jel” from the HEWs during their home visits. The study done in Ethiopia also stated that even though the “Yimserach jel” was manufactured locally, its availability was limited outside of facilities [37].

This study also found that, the HCs were not equipped with regard to the required competency and required essential medicines, supplies and equipment to manage preterm labour. As a result, the health centres role limited to rereferral of expected cases to hospital, mainly without providing he recommended pre-referral treatment. At the current standards and health centre readiness limitation, participants could not manage women presenting with preterm labour as outlined by WHO [26]. As stipulated in the national obstetrics management protocol for health centres in Ethiopia, when preterm labour is diagnosed, the protocols recommend urgent referral after pre-referral care is provided. Dexamethasone injection is one of the pre-referral management. However, most of the time, the respondents acknowledged that it is not available in the health centre. Only in the case of imminent delivery, it is recommended attend the delivery at the health centres where hands-on skills are lacking [38].

Overall, the findings are expected to contribute to revisiting the implementation strength of the high impact newborn healthcare interventions in the primary healthcare and updating of strategies, job-aids and implementation manuals by regional health bureaus and the ministry of health.

## Conclusion

As per the experience of healthcare professionals in primary healthcare, they are facing health system related barriers to providing high-impact interventions and quality of care for newborns during labour and delivery and in the early postnatal period. The quality of newborn healthcare provision in North-West Ethiopia should be enhanced to improve the quality of life and reduce neonatal mortality and morbidity. In addition, the health systems related constraints at health centres and health posts levels should be fixed to provide the required service for newborns.

### Recommendations

- The primary healthcare facilities play a major role in the provision of essential services for newborns in the critical periods, including during labor and birth, immediately after birth and in the early postnatal care period. This requires allocation of adequate resources to tackle health facilities readiness related bottlenecks, such as the frequent stock out or lack of essential supplies, equipment, and medicines, lack of proper space for the service provision, not systematic replenishing of the revised job-aids and maintenance of medical equipment, poor infection prevention including water and sanitation in the maternity wards and newborn corners.
- The Ministry of health in collaboration with developing partners, professional associations, and local universities, should organize the low-dose high frequency onsite hands-on training for the clinicians to improve their skills to provide the quality of newborn care services, at least every year.
- The training needs to be augmented by clinical mentorship or a systematic programme specific supportive supervision visits to the respective healthcare facilities. This will help to build clinicians and healthcare providers confidence and improve their competency on the quality of newborn healthcare services provision and ensure adherence on the use of job-aids and service delivery standards.
- The local authorities and the PHCUs shall provide attention and focus to improving the quality of newborn health services during labour, delivery and in the early neonatal period.
- Improving the coverage and quality of PNC home visits at the community level should be the major tasks by HEWs. It should be a targeted visit with adequate preparation including essential checklist and supplies; so that, the HEWs can provide the routine newborn care and early detect the premature, low birth weight or babies’ signs of infection.

## Data Availability

It can be obtained from the corresponding author.

## Abbreviations

FDG: Focus Group Discussion
HCs: Health Centres
HEWs: Health Extesion Workers
HWs: Health Workers
KMC: Kangaroo Mother Care
PHCUs: Primary Healthcare Units
PNC: Postnatal Care
